# Changes in Motor Skill Proficiency After Equine-Assisted Activities and Brain-Building Tasks in Youth With Neurodevelopmental Disorders

**DOI:** 10.3389/fvets.2020.00022

**Published:** 2020-01-31

**Authors:** B. Rhett Rigby, Ronald W. Davis, Melissa D. Bittner, Robin W. Harwell, Eileen J. Leek, Geoben A. Johnson, David L. Nichols

**Affiliations:** ^1^School of Health Promotion and Kinesiology, Texas Woman's University, Denton, TX, United States; ^2^Department of Kinesiology, California State University Long Beach, Long Beach, CA, United States; ^3^Mangait Therapeutic Horsemanship, McKinney, TX, United States

**Keywords:** adolescents, children, equine-assisted activities, motor proficiency, neurodevelopmental, plasticity, therapeutic horseback riding

## Abstract

There is a lack of current research to support the efficacy of a combination of equine-assisted activities (EAA) and brain building activities to influence motor skill competencies in youth with neurodevelopmental disorders (ND). The primary objective of this study was to quantify changes in motor skill proficiency before and after 8 weeks of EAA and brain-building activities in youth with ND. A secondary objective was to quantify changes in motor skill proficiency before and after 1 year of EAA and brain-building activities in youth with ND. Twenty-five youth completed the same 32-week protocol that was separated into 4, 8-week blocks, in the following order: (1) control; (2) EAA-only; (3) washout; (4) GaitWay block (EAA and brain building activities). Before and after each block, motor skills were assessed using the Short Form of the Bruininks-Oseretsky Test of Motor Proficiency-Version 2 (BOT-2). Seven youth continued with the GaitWay intervention for one additional year, and the BOT-2 Short Form was also administered following this intervention. A repeated-measures analysis-of-variance was performed to compare BOT-2 subtest and overall scores between interventions with a significance of 0.05. Manual dexterity was higher at Post-Washout [3.3 (2.4)] vs. Pre-Control [2.2 (2.1); *p* = 0.018] and Post-Control [2.6 (2.0); *p* = 0.024], and at Post-GaitWay vs. Pre-Control [3.2 (2.4) vs. 2.2 (2.1); *p* = 0.037]. Upper-limb coordination was higher at Post-GaitWay vs. Post-Control [6.0 (4.1) vs. 3.9 (3.8); *p* = 0.050]. When compared to Pre-Control [3.2 (3.0)], strength was higher at Post-EAA [4.9 (3.5); *p* = 0.028] and at Post-GaitWay [5.2 (2.9); *p* = 0.015]. Overall scores were higher at Post-GaitWay [39.1 (22.2)] when compared to Pre-Control [32.4 (21.6); *p* = 0.003] and Post-Control [32.5 (21.9); *p* = 0.009]. Additionally, motor skills were maintained for 1 year following the Post-GaitWay testing session among seven participants. This is the first known study to include and demonstrate the short-term and long-term effects of a combination of EAA and brain building activities with motor proficiency in youth with ND.

**Clinical Trial Registration:** Motor Skill Proficiency After Equine-Assisted Activities and Brain-building Tasks; www.ClinicalTrials.gov, identifier: NCT04158960.

## Introduction

Neurodevelopmental disorders (ND) are conditions characterized by developmental deficits and impairments in language and speech, cognition, behavior, and motor skills (1). Associated with the dysfunction of the brain and central nervous system, children and adolescents (youth) with ND typically exhibit impairments related to personal, social, and academic performance and functioning (2). According to the *Diagnostic and Statistical Manual of Mental Disorders* (DSM-5), examples of ND include attention deficit hyperactivity disorder (ADHD), autism spectrum disorder (ASD), intellectual developmental disorder (IDD), global developmental delays, disorders associated with communication and speech, learning disorders (LD), and mood disorders (2). In many cases, there are several co-diagnoses related to abnormal neurodevelopment for a particular youth (2). In the United States, ~1 in 6 children have some developmental disability (i.e., LD, ADHD, ASD, other developmental delays) (3). For youth referred to mental health services, prevalence rates of ND can be as high as 55.5%, with the majority of diagnoses occurring with boys (4).

The increasing incidence of ND with other co-diagnoses, particularly metabolic diseases such as obesity, is a significant public health issue (5, 6). Children with ASD are ~1.5 times more likely to be overweight or obese compared to their typically developing peers (7). Prevalence rates of children with ADHD and obesity can range between 20 and 50%, depending on the population and location (8, 9). Among children ages 11–17 years, ADHD is twice as common among those who are overweight or obese when compared to children who are normal weight (10). Obesity and its causal environmental factors are likely to track into adulthood as well, depending on the age of onset, obesity severity, and whether parents are obese (11, 12). A two-fold increase in obesity rates exist among adults with ADHD who were obese in childhood compared to those without ADHD (13).

One environmental factor that can have a substantial impact on obesity, and overall health, is sedentary behavior. It has recently been reported that children spend ~7.7 h per day being sedentary (14). Indeed, low levels of physical activity and high levels of sedentariness are associated with an increased risk of obesity and cardiometabolic disease, and decreased cognitive processing and psychosocial well-being in youth (15–17). This is particularly significant for those with ND, as adolescents with ND are more likely to be sedentary and less likely to exercise or participate in organized sports when compared to their age-matched peers without ND (18).

Physical activity is an effective, cost-efficient method to combat sedentary behaviors, obesity, and psychosocial health in those with ND. An inverse relationship exists between amounts of physical activity and body weight and adiposity in youth ages 3–17 years (6). Regular exercise can also improve cognitive function in children and adolescents ages 6–13 years (6). One factor that could affect regular physical activity habits and programming, including exercise mode, frequency, intensity, and duration, is motor function. Participation in physical activity is correlated with the ability to efficiently perform motor skills (19). Positive relationships exist between motor skill competency, likelihood to engage in physical activity, and health-related fitness (20, 21). Organized physical activity programs that incorporate group leaders and other peers can mitigate setbacks in development and improve motor and non-motor skills in those with ND (22). Examples of these programs include tennis, martial arts, football, and horseback riding (23). Specifically, equine-assisted activities and therapies (EAAT), which were established and are currently regulated by the Professional Association of Therapeutic Horsemanship (PATH), have gained recent attention as an effective alternative modality to improve motor skills and gross motor function in youth with disabilities (24).

Equine-assisted activities (EAA), or therapeutic horseback riding, involves the teaching of specific riding skills (e.g., mounting and dismounting, guiding the horse) by non-licensed professionals to improve learning in persons with various disorders and disabilities (24). This programming differs from the rehabilitation-focused form of EAAT, equine-assisted therapies (EAT). Traditionally called hippotherapy, licensed physical, occupational, or speech therapists use the movement of the horse to improve the functional and psychosocial health of the participant during EAT sessions (24). With both EAA and EAT, the three-dimensional, rhythmic movements of the horse force the rider to adjust to these movements and activate musculature in the lower torso and pelvis (25). By generating movements at the rider's pelvis that resemble those essential for ambulation, improvements in gross motor function can occur (26, 27). There is evidence of improvements in motor skills following EAAT interventions in youth with ND. Strength and balance improved after 10 weeks of hippotherapy in adolescents with IDD (28). In children with ADHD, manual dexterity and bilateral coordination improved after 12 weeks of EAAT (29). Twelve weeks of hippotherapy elicited improvements in postural sway and balance in children with ASD (30). Gross motor function, as measured by the gross motor function measure (GMFM), was improved after 8 weeks of EAAT in youth with psychomotor impairments [e.g., developmental delays (31)].

Equine-assisted activities are typically conducted at PATH-accredited therapeutic riding centers. Many of these centers across the United States are beginning to adopt a program that combines EAA with brain-building activities (i.e., tasks that are used to improve the brain's ability to process information that comes into the body along the primary sensory pathways, including auditory, visual, and vestibular pathways) to enhance motor proficiency in youth with ND. One such center to adopt this approach is ManeGait Therapeutic Horsemanship (McKinney, TX). Termed “The GaitWay to the Brain” program, participants undergo EAA along with a number of brain-building activities completed off of the horse, both on-site at ManeGait Therapeutic Horsemanship. This approach differs from the more traditional therapeutic model of teaching, or re-teaching toward a goal, that is practiced over time and with support. The goal in the GaitWay program is not teaching or re-teaching a selected daily activity (e.g., building vocabulary, dressing self, managing stairs, handwriting, or math problems), as in traditional therapies, but in completing the tasks consistently and as directed. When these activities are performed consistently over time, the participant's brain may be able to process sensory information more readily. The brain may also be able to integrate this sensory information more accurately and with less effort, improving the brain-body's ability to attain and sustain attention that supports learning and function (32).

As prevalence rates of ND continue to increase, the need to address the associated motor deficits in youth with ND is critical, particularly due to the increased risk of other chronic diseases that can impair health in this population. Alternative modes of physical activity may be useful tools to mitigate the deficits in motor development in those with ND. To date, there is no current research to support the efficacy of a combination of EAA and brain-building activities to influence motor skill competencies in youth with ND. The primary purpose of this study was to quantify changes in motor skill proficiency before and after 8 weeks of EAA and brain-building activities in youth with ND (primary analyses). A secondary purpose was to quantify changes in motor skill proficiency before and after 1 year of EAA and brain-building activities with a subset of youth with ND who participated in the primary analyses (follow-up analyses). Our hypotheses were: (1) 8 weeks of a combination of EAA and brain-building activities would increase motor skill proficiency in a greater manner when compared to performing 8 weeks of EAA alone; (2) motor proficiency would be increased following 1 year of both EAA and brain-building activities compared to baseline (which served as the end of the primary analyses and the start of the follow-up analyses).

## Materials and Methods

### Primary Analyses

#### Participants

Thirty-one children and adolescents, ages 5–16 years, diagnosed with some neurodevelopmental disorder as a primary diagnosis, were initially recruited from a waitlist through ManeGait Therapeutic Horsemanship. All participant diagnoses were made by a physician, psychiatrist, or psychologist prior to study enrollment. We received confirmation of these diagnoses at the time of the participant's screening. Twenty-five youth (13 males, 12 females) completed all procedures. A reason for not completing the study included a perfect score on the motor proficiency test conducted during the first testing session. Other reasons included a fearfulness of horses and scheduling conflicts, both of which affected regular attendance at testing and intervention sessions and overall compliance. The diagnoses and associated prevalence of all participants can be found in Table 1. The most commonly reported diagnoses were ADHD and ASD. Many of the participants had several co-diagnoses.

**Table 1:** Participant diagnoses and prevalence.

**Diagnosis**	**Diagnosis**
Attention deficit Hyperactivity disorder (*n* = 9)	Pervasive developmental disorder – Not otherwise specified (*n* = 1)
Autism spectrum disorder (*n* = 9)	Dyslexia (*n* = 1)
Intellectual developmental disorder (*n* = 6)	General anxiety disorder (*n* = 1)
Sensory processing disorder (*n* = 5)	Mood disorder (*n* = 1)
Global developmental delay (*n* = 5)	Oppositional defiance disorder (*n* = 1)
Speech impairment (*n* = 3)	Williams syndrome (*n* = 1)
Hypotonia (*n* = 2)	Gross motor delay (*n* = 1)
Audio processing disorder (*n* = 2)	Reactive attachment disorder (*n* = 1)

Characteristics of the final 25 participants can be found in Table 2. On average, the body mass index of the participants is classified as “healthy weight” according to data available from the Centers for Disease Control (33).

**Table 2:** Participant characteristics (primary analyses).

**Variable**	**Mean (*SD*)**	**Min**	**Max**
Age (years)	9.7 (2.6)	5.0	16.0
Height (cm)	136.2 (15.2)	106.7	165.1
Weight (kg)	34.1 (11.3)	19.1	58.1
BMI (kg/m^2^)	18.2 (5.0)	11.3	33.7

*Descriptive statistics of 25 youth (13 males, 12 females) with neurodevelopmental disorders. BMI, body mass index*.

Additionally, we screened the youth to include those who: (1) had the ability to follow verbal directions; (2) did not have a perfect score on the motor proficiency test at the first testing session; (3) were without seizures within the past 6 months controlled by medication; (4) without a known allergy to horses; (5) free from any surgical procedures performed within the 6 months; (6) were without regular horseback riding experience of any kind during the past year. All participants were cleared by a medical professional to participate in the study. All youth (and their caregivers) signed a university-approved written informed consent, in accordance with the Declaration of Helsinki, and a photo/video release form before the study began. This study was carried out in accordance with the recommendations of, and was approved by, the first author's Institutional Review Board. The attending veterinarian for the first author's institution reviewed the methodology for this study and made the determination that a formal proposal to the university's Institutional Animal Care and Use Committee was not needed. The equines in this study were well-trained for, and experienced in, EAA. Also, the equines in this study were provided with routine veterinary medical care and were limited in the number of EAA sessions performed daily. No research was conducted directly with the equines in the current study (including no medical or experimental procedures and no euthanasia), and the equines were used in accordance with the usual policies, procedures, and practices of ManeGait Therapeutic Horsemanship.

#### Experimental Procedure Overview

The experimental protocol included an entry session and five testing sessions, each separated by an 8-week time period. All testing was conducted by the same trained personnel. All youth participated in EAA and the GaitWay program at ManeGait Therapeutic Horsemanship. During the riding sessions, each participant was assigned to a single horse leader and certified riding instructor, with additional horse handlers and side walkers. During the GaitWay sessions, each participant was assigned to the same licensed speech therapist.

The participants were instructed to maintain their current course of medications and any additional, outside therapies. The participants were also asked to not begin any new therapeutic intervention (e.g., medication, physical therapy) during the study. The caregivers were also asked to inform the researchers of any major changes in diet, sleep, or daily stressors. Although there were occasional questions from caregivers regarding switching medications or starting a new experimental therapy, all participants and their caregivers complied with study parameters.

**Figure 1:**
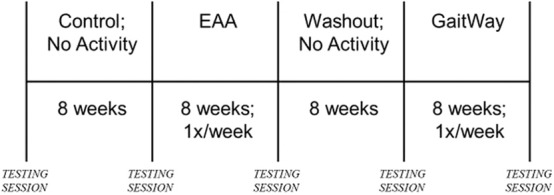
Study timeline.

The protocol consisted of 4, 8-week time blocks (see Figure 1). The first 8-week block was a control period, in which the participants were not required to visit ManeGait. However, participants were asked not to engage in horseback riding of any kind (e.g., recreational, therapeutic). In the second 8-week block, the youth performed EAA only at ManeGait, and did not participate in the GaitWay program. The third 8-week block was a washout period and replicated the first 8-week control block with participant requirements. In the fourth and final 8-week block, the youth participated in the GaitWay sessions. In these sessions, the brain-building activities followed horseback riding during the same visit. During blocks 2 and 4, all participants attended ManeGait once per week. Equine-assisted activities were administered once per week, with each session lasting 45–60 min. The GaitWay sessions were administered face-to-face once per week and each session was 90 min in duration.

#### Testing Sessions

A total of five testing sessions occurred (see Figure 1). Each testing session was held as follows: (1) before block 1 (Pre-Control); (2) after block 1 (Post-Control); (3) after block 2 (Post-EAA); (4) after block 3 (Post-Washout); (5) after block 4 (Post-GaitWay). There was no additional time scheduled between blocks. Therefore, for the middle three testing sessions, each session occurred at the end of a block and the start of the subsequent block (e.g., the Post-Control testing session occurred at the end of the control block and the start of the EAA-only block). Each testing session occurred within 48 h after the end of the last session in a block, with the exception of the first testing session, which was held no more than 48 h before the start of the first block. The blocks were not randomized due to necessary scheduling of sessions at ManeGait Therapeutic Horsemanship. Thus, each participant followed the same order of interventions and testing sessions.

At the start of each testing session, verbal assent was obtained from the participants before the day's procedures. The caregivers were reminded that all testing was filmed using a video camera (to verify scoring for each tester). Prior to the start of data collection, extra personnel and any item that could be removed from the room that was deemed to be a distraction (e.g., various pieces of equipment, toys) was placed in another location, out of the vision of the participant. The participants motor skill proficiency was then assessed using the Short Form of the Bruininks-Oseretsky Test of Motor Proficiency-Version 2 (BOT-2). Derived from the BOT-2 Complete Form, this shorter testing battery was designed to assess motor skill competence for those 4–21 years old with mild to severe motor problems, and contains 14 activities within the following subtests: (1) fine motor precision (drawing a line through a path, folding paper); (2) fine motor integration (copying shapes); (3) manual dexterity (transferring pennies); (4) bilateral coordination (tapping feet and fingers, jumping in place with unilateral synchronization); (5) balance (walking on a line, standing on one leg on a balance beam); (6) running speed and agility (one-legged stationary hop); (7) upper-limb coordination (dropping and catching ball, dribbling a ball with alternate hands); (8) strength (knee push-ups, sit-ups) (34, 35). A total score was calculated from the sum of all subtests.

A score for each subtest was determined from the performance of the tasks within each category. A total score was then calculated based on the sum of scores from all subtests. A high correlation (*r* = 0.80) exists between the BOT-2 Short Form and the BOT-2 Complete Form, indicating strong content validity (34, 35). In middle-age school youth (the primary age group recruited for this study), results of the BOT-2 Short Form are not different from the BOT-2 Complete Form and is a useful tool to assess motor competencies in youth with motor delays (36). Indeed, when using the BOT-2 Short Form with knee-pushups (vs. full push-ups) in the Strength subtest, a very high inter-rater reliability of *r* = 0.98 and a test–retest reliability of *r* ≥ 0.80 exists (34, 35). There is also reasonable proof of convergent validity with Körperkoordinationstest für Kinder (KTK), a validated test used to assess motor proficiency in children and adolescents with moderate to severe motor skill deficiencies (37). Total time administration for each testing session was 25–35 min for each participant. Each participant was evaluated by the research personnel for all testing sessions.

At the start of the 3rd (Post-EAA) and last (Post-GaitWay) testing session, caregivers of each participant were asked to verbally report any changes observed with their child or adolescent over the previous 8 weeks. These anecdotal reports provided valuable insight into the perceived effects, including those outside the scope of the motor proficiency assessment, of the interventions. These reports were transcribed by hand and recorded. All statements remained confidential.

#### Equine-Assisted Activity Intervention

Equine-assisted activities were performed alone in the second 8-week block and with brain-building activities as part of the GaitWay program in the last 8-week block. At the start of each EAA session, the caregivers and participants were familiarized with the EAA session procedures. An effort was made to keep the horse, saddle, and activities performed on top of the horse consistent between sessions for a particular youth participant. Some horses were chosen compared to others due to their gait patterns (e.g., choppy vs. smooth gait), with their gait correlating to the level of disability for each participant. Different saddles were also used (e.g., Dressage saddle, Western saddle) for varying levels of stability. ManeGait Therapeutic Horsemanship is accredited by the Professional Association of Therapeutic Horsemanship International as a premier center. All sessions were conducted under the direction and supervision of certified therapeutic riding instructors.

Participants gave verbal assent at the start of each EAA session. Because each participant differed in functional abilities, a specific protocol was created for each youth. However, there were some common activities performed across sessions for all participants. Before riding began, warm-up activities were performed, which included gross body movements (e.g., arm circles, body-weight squats). Several participants would then practice mounting and dismounting on a stationary barrel before attempting the same process on their actual horse. Once warm-up activities were completed, participants would (independently) choose a helmet to wear and affix it to their head. Tack, or additional equipment used during the EAA session (e.g., reins, harnesses, bits, stirrups), was then gathered by the youth and taken to the arena. In each session, but particularly emphasized in the first few sessions, participants were taught: (1) the anatomical parts of the horse, including the head, mane, and tail; (2) how to ride, mount, and dismount; (3) where to touch the horse when mounted or dismounted; (4) the names and function of tack equipment. A point of emphasis given by the instructors and volunteers present to the youth was to trust the horse and all helpers, in order to have the EAA sessions be completed without numerous interruptions. The participant would then attempt to mount the horse while standing on a nearby ramp. Some participants needed assistance, but a goal for most of the participants was to execute the mounting process without assistance.

Once on the horse, the instructors would emphasize a proper sequencing of events in order for the youth to initiate and maintain horse movement. This included only moving the horse when certain words were spoken, focusing on heel position, awareness of hand position on the saddle, keeping the head straight and looking forward while maintaining eye contact with the leader, and managing varying postural changes. Participants were reminded to sit upright, particularly while the horse was trotting, but were also instructed to assume other postures, including a two-point stance (i.e., forward leaning) and posting (i.e., rising out of the saddle every other stride during a trot). A trotting cadence was initiated by the participants using their legs and word commands. While trotting, the youth were instructed to steer the horse through a variety of cone patterns, which included some turning. Other activities including guiding the horse through a Figure-8 pattern and on a trail ride. While the horse was in motion, some additional activities, including functional reach exercises, were performed by the participants. Constant feedback was requested by the instructor and volunteers once the youth mounted the horse. Although it varied for each participant, some of the common feedback modes were answers to verbal questions and commands, making eye contact with the instructor, and using high-fives when accomplishing a task. Once the riding was completed, the participant was instructed to dismount the horse and replace the helmet and tack in a nearby room.

#### GaitWay to the Brain Intervention

The GaitWay intervention included EAA and brain-building activities. Similar to the procedures associated with EAA, verbal assent was given by all participants prior to the execution of any brain-building activities. These activities completed during the GaitWay sessions were held in a structure on the grounds of ManeGait, called the GaitHouse. The GaitHouse is several rooms in a remodeled mobile home. Once in the GaitHouse, the speech therapist moved the rider through a variety of sensory stations and multiple activities. The riders were seen individually if possible. If scheduling required two participants simultaneously (which was the maximum number that could be scheduled in the GaitHouse), the speech therapist would work with one participant, while a trained staff worked with the other participant following the protocol designed by the therapist. The close proximity of the therapist and staff member in the GaitHouse allowed for the therapist to observe and intercede if necessary. The schedule of youth during the GaitWay sessions was maintained throughout the study, whether participants were seen individually or with another participant simultaneously. Similar to the EAA sessions, a specific protocol was created for each youth.

The youth were first assessed by a licensed speech therapist to determine the sensory input channels in which they were struggling, as well as their ability to integrate this sensory information among the various sensory pathways. Then tools and activities were selected to follow in the GaitHouse and for use at home for carryover training. The participant and caregivers were educated regarding the assessment findings, and then educated on how to use the tools and how to complete the activities daily at home. The therapist requested that participants complete home activities daily. If this was not possible, participants were instructed to complete the home activities a minimum of 2 to 3 times per week.

Visual-vestibular integration was targeted using either one or more of the following: (1) an “astronaut board”; (2) a platform swing; (3) a lycra swing; (4) a barber chair that allowed for the rider to spin in a supine position. After this activation, deep touch was applied to the participant to organize the brain-body. Having the youth follow a visual target across the visual midline, and then converging and diverging on the object, completed the activity. Modulated music (i.e., music that has been filtered by frequencies) and music using binaural beats (for calming and organizing the brain-body connection) was also provided via headphones placed on the participant. This equipment was used first in the GaitHouse during the assessment for music selection, and then taken home for listening in one to two 30-min daily sessions. Other activities included several selected activities from Brain Gym, including the Double Doodle (i.e., a bilateral drawing exercise) and P.A.C.E. (i.e., a 4-step routine of brain-body movements designed to move the participant into a “ready” state for learning) (38). Each participant verbalized a positive statement targeting a self-selected or caregiver selected “goal,” which was followed by these integrative movements of P.A.C.E.

#### Statistical Analyses

All participants attempted all testing sessions. Although some participants scored a “zero” on the BOT-2 Short Form in some testing sessions, these scores were included in the analysis. The independent variable was intervention. The dependent variables included scores on each subtest of the BOT-2 Short Form and an overall score. Multiple repeated-measures analysis of variance (ANOVAs) were performed to compare BOT-2 Short Form scores between time points. Bonferroni *post-hoc* tests were used to follow-up significant differences in scores. Effect sizes (eta squared) were also calculated for all variables. Results were analyzed using SPSS v.24 (IBM Inc., Armonk, NY) with a significance level of 0.05. A correction for the *p*-values was not implemented for the multiple tests that were conducted. This is noted in the limitations section of the Discussion. An a priori power analysis was also performed using G^*^Power 3.1.9 (Düsseldorf, Germany). Using a moderate effect size of 0.25 and a power of 0.80, a sample size of 21 was required.

### Follow-Up Analyses

Seven children and adolescents, ages 6–15 years, diagnosed with some neurodevelopmental disorder as a primary diagnosis, were initially recruited from the primary analyses to continue to participate in the GaitWay program at ManeGait Therapeutic Horsemanship for one additional year. All participants (2 males, 5 females) completed all intervention procedures. Common diagnoses among these participants were ADHD and ASD (*n* = 3 each). Most of the participants possessed several co-diagnoses, as other diagnoses included IDD, sensory processing disorder, mood disorder, dyslexia, audio processing disorder, oppositional defiance disorder, general anxiety disorder, and pervasive developmental disorder-not otherwise specified (*n* = 1 each). Characteristics of the seven participants can be found in Table 3. Because the youth were participants in the primary analyses, similar inclusion criteria were met for the follow-up analyses. All participants were cleared by a medical professional to participate in the study. All youth (and their caregivers) signed a university-approved written informed consent and a photo/video release form before the analyses began.

**Table 3:** Participant characteristics (follow-up analyses).

**Variable**	**Mean (*SD*)**	**Min**	**Max**
Age (years)	11.5 (2.8)	6.0	15.0
Height (cm)	139.1 (16.0)	116.8	157.5
Weight (kg)	40.0 (12.7)	20.9	54.4
BMI (kg/m^2^)	20.7 (6.5)	13.8	33.9

*Descriptive statistics of 8 youth (3 males, 5 females) with neurodevelopmental disorders. BMI, body mass index*.

The experimental protocol included one additional testing session which followed a 1-year intervention (GaitWay). During the EAA sessions, each participant was assigned to a single horse leader and certified riding instructor, with additional horse handlers and side walkers. During the GaitWay sessions, each participant was assigned to the same licensed speech therapist. Similar to the primary analyses, the participants were instructed to maintain their current course of medications and any additional, outside therapies. No new therapeutic intervention (e.g., medication, physical therapy) was begun during the study. Similar procedures were followed during the EAA and brain-building activity sessions.

With regard to the statistical analyses, the independent variable was intervention and the dependent variable was an overall score on the BOT-2 Short Form. A repeated-measures analysis of variance was performed to compare overall BOT-2 Short Form scores between time points (i.e., Post-Washout, Post-GaitWay, and 1-Year). Bonferroni *post-hoc* tests were used to follow-up significant differences in scores. Effect sizes (partial eta squared) were also calculated. Results were analyzed using SPSS v.24 (IBM Inc., Armonk, NY) with a significance level of 0.05. With only seven participants, the BOT-2 Short Form subtests were not compared with this analyses, as multiple ANOVAs would reduce the statistical power.

## Results

### Primary Analyses

Motor skill scores with associated effect sizes can be found in Table 4. A significant effect for the intervention was found with manual dexterity [*F*_(2.81,67.38)_ = 6.996, *p* < 0.001], bilateral coordination [*F*_(4,96)_ = 2.703, *p* = 0.035], upper-limb coordination [*F*_(4,96)_ = 3.326, *p* = 0.013], and strength [*F*_(3.07,73.74_) = 6.017, *p* = 0.001]. *Post-hoc* analyses revealed that manual dexterity was higher at Post-Washout vs. Pre-Control (*p* = 0.018) and Post-Control (*p* = 0.024), and at Post-GaitWay vs. Pre-Control (*p* = 0.037). Upper-limb coordination was higher Post-GaitWay vs. Post-Control (*p* = 0.050). When compared to Pre-Control, strength was higher at Post-EAA (*p* = 0.028) and at Post-GaitWay (*p* = 0.015). No differences between time points were found with bilateral coordination using *post-hoc* analyses. All other subscale scores were not different (*p* > 0.05). Effect sizes (using eta squared) for all dependent variables ranged from 0.011 to 0.245.

**Table 4:** Subtest scores on the BOT-2 Short Form at specific time points.

**Subtest**	**Max**	**Pre-C**	**Post-C**	**Post-EAA**	**Post-WO**	**Post-GW**	**${\text{η}}_{\text{P}}^{2}$**
Fine motor precision	14	6.1 (4.9)^a^	6.0 (4.7)^a^	6.3 (4.8)^a^	6.2 (4.5)^a^	6.4 (5.2)^a^	0.011
Fine motor integration	10	4.4 (3.2)^a^	4.2 (3.0)^a^	4.4 (2.9)^a^	5.0 (3.3)^a^	4.8 (3.1)^a^	0.071
Manual dexterity^a^	9	2.2 (2.1)^a^	2.6 (2.0)^ab^	2.9 (2.0)^ac^	3.3 (2.4)^c^	3.2 (2.4)^bc^	0.226
Bilateral coordination^a^	7	3.6 (2.9)^a^	3.8 (2.7)^a^	4.2 (3.1)^a^	4.2 (2.9)^a^	4.7 (2.9)^a^	0.101
Balance	8	4.0 (3.1)^a^	3.9 (2.9)^a^	3.4 (2.8)^a^	4.1 (2.7)^a^	4.3 (2.7)^a^	0.093
Running speed and agility	10	4.1 (3.5)^a^	4.0 (3.4)^a^	4.0 (3.2)^a^	4.1 (3.3)^a^	4.7 (3.1)^a^	0.038
Upper-limb coordination^a^	12	4.7 (4.4)^ab^	3.9 (3.8)^a^	5.2 (4.2)^ab^	4.7 (4.1)^ab^	6.0 (4.1)^b^	0.122
Strength (w/ knee-pushups)^a^	18	3.2 (3.0)^a^	4.1 (3.1)^ab^	4.9 (3.5)^b^	4.6 (3.2)^ab^	5.2 (2.9)^b^	0.200

*Mean (SD) reported. Higher scores denote improved performance. Values with the same superscript are not statistically different*.

a *Significant effect for intervention. BOT-2, Bruininks-Oseretsky Test of Motor Proficiency-Version 2; Max, maximum possible score; Pre-C, Pre-Control period; Post-C, Post-Control period; Post-EAA, Post-Equine-Assisted Activities period; Post-WO, Post-Washout period; Post-GW, Post-GaitWay period; $\eta_{P}^{2}$, effect size*.

**Figure 2:**
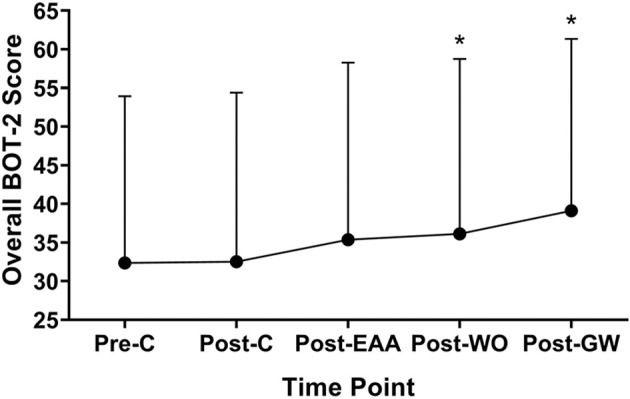
Overall scores on the Short Form of the Bruininks-Oseretsky Test of Motor Proficiency-Version 2 (BOT-2) between time points *(n* = 25). *Significantly greater than Pre-C *(p* < 0.05). Pre-C, Pre-Control time point; Post-C, Post-Control time point; Post-EAA, Post-Equine-Assisted Activities time point; Post-WO, Post-Washout time point; Post-OW, Post-GaitWay time point.

**Figure 3:**
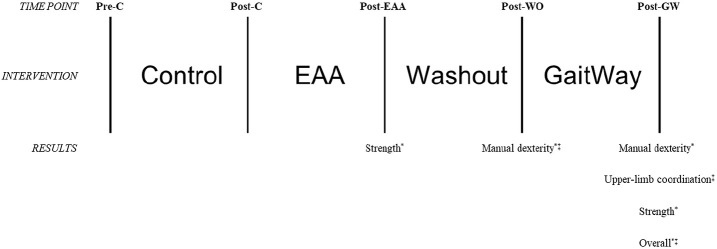
Visual representation of changes in motor skills as assessed using the Short Form of the Bruininks-Oseretsky Test of Motor Proficiency-Version 2 (BOT-2) between time points in the primary analyses *(n* = 25). *Significantly greater than Pre-C *(p* < 0.05); ^‡^significantly greater than Post-C *(p* < 0.05). Pre-C, Pre-Control time point; Post-C, Post-Control time point; Post-EAA, Post-Equine-Assisted Activities time point; Post-WO, Post-Washout time point; Post-GW, Post-GaitWay time point.

Overall motor skill scores from the BOT-2 Short Form can be found in Figure 2. A significant effect for the intervention was found, *F*_(2.85,68.41)_ = 7.796, *p* < 0.001. Scores were higher at Post-GaitWay when compared to Pre-Control [39.1 (22.2) vs. 32.4 (21.6); *p* = 0.003] and Post-Control [39.1 (22.2) vs. 32.5 (21.9); *p* = 0.009]. No significant differences were found between any other time points. An effect size of $\eta_{\text{P}}^{2}$ = 0.245 was calculated for these time points. Statistically relevant results from the primary analyses are summarized in Figure 3.

Anecdotal responses from caregivers, given at the start of 3rd and last testing sessions, are provided in Table 5. Improvements in balance and posture were the most common responses following both the EAA-only and GaitWay interventions.

**Table 5:** Anecdotal responses from caregivers of participants following the EAA-only intervention and the GaitWay intervention.

**At Post-EAA**	
Improved balance/posture (*n* = 11)	More calm demeanor (*n* = 1)
Improved core strength (*n* = 3)	Reduced behavioral outbursts at school (*n* = 1)
**At Post-GW**	
Improved balance/posture (*n* = 14)	Improved eye tracking (*n* = 1)
Improved verbal, written compliance (*n* = 9)	Better understanding of complex directions (*n* = 1)
More regulated/calm (*n* = 9)	Improved abstract thinking (*n* = 1)
Improved limb coordination (*n* = 8)	Shift to more concrete objects in drawings (*n* = 1)
Improved overall academic performance (*n* = 7)	Improved multi-tasking (*n* = 1)
Improvement in vision/focus (*n* = 7)	More independent (*n* = 1)
Increased desire to initiate verbal conversations (*n* = 7)	Less interaction with an imaginary friend (*n* = 1)
Increased confidence (*n* = 6)	More time spent engaging with friends (*n* = 1)
Improved core strength (*n* = 5)	No incidence of seizures with a previous history (*n* = 1)
Started reading or enhanced reading skills (*n* = 5)	Taking more accountability for actions (*n* = 1)
More affectionate and empathetic (*n* = 5)	Improved stamina while riding (*n* = 1)
Improved handwriting (*n* = 4)	Increased desire to participate in sports (*n* = 1)
Improved memory (*n* = 4)	Improved eating habits (*n* = 1)
Improved sleep (*n* = 3)	Improved relationship with siblings (*n* = 1)
Improved ability to spell and organize sentences (*n* = 2)	Improved toilet training (*n* = 1)

*Each response denotes number of participants (n). EAA, equine-assisted activities; Post-EAA, Post-Equine-Assisted Activities time point; Post-GW, Post-GaitWay time point*.

### Follow-Up Analyses

**Figure 4:**
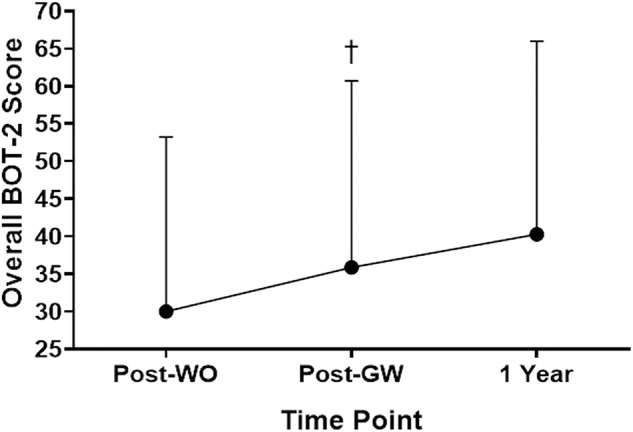
Overall scores on the Short Form of the Bruininks-Oseretsky Test of Motor Proficiency-Version 2 (BOT-2) between time points *(n* = 7). †Significantly greater than Post-WO (*p* = 0.035). Post-WO, Post-Washout time point; Post-GW, Post-GaitWay time point; 1 Year, 1 year time point following the Post-GaitWay time point.

Overall motor skill scores from the BOT-2 Short Form for the 1-year follow up analyses can be found in Figure 4. A significant main effect for the intervention was found, *F*_(1.18,7.07)_ = 8.343, *p* = 0.021. Scores were higher at Post-GaitWay when compared to Post-Washout [35.9 (24.9) vs. 30.0 (23.2); *p* = 0.035]. No significant differences were found between any other time points. An effect size of $\eta_{\text{P}}^{2}$ = 0.582 was calculated for these time points.

Anecdotal responses from caregivers, given at the start of 1-year follow-up testing session, are provided in Table 6. Enhanced coping skills and improvements in balance and posture were the most common responses following the 1-year GaitWay intervention.

**Table 6:** Anecdotal responses from caregivers of participants following the 1-year GaitWay intervention.

Improved balance/posture (*n* = 5)	Improved memory (*n* = 1)
Enhanced coping skills (*n* = 5)	Improved focus (*n* = 1)
Improved overall academic performance (*n* = 5)	Improved hand/eye coordination (*n* = 1)
Increased confidence (*n* = 2)	Improved handwriting (*n* = 1)
Learned how to ride a bicycle (*n* = 1)	Can communicate details in pictures (*n* = 1)
Increased desire to initiate verbal conversations (*n* = 1)	Enhanced reading skills (*n* = 1)
Improved eating habits (*n* = 1)	Improved math skills (*n* = 1)
Increased desire to be active (*n* = 1)	

*Each response denotes number of participants (n)*.

## Discussion

The primary purpose of this study was to quantify changes in motor skill proficiency before and after 8 weeks of EAA and brain-building activities in youth with ND (primary analyses). With regard to the subtest scores, differences were observed with manual dexterity, upper-limb coordination, and strength. Manual dexterity was higher at Post-washout vs. Pre-Control and Post-Control, and at Post-GaitWay vs. Pre-Control. Upper-limb coordination was higher Post-GaitWay vs. Post-Control. When compared to Pre-Control, strength was higher at Post-EAA and at Post-GaitWay. When comparing overall scores, Post-GaitWay scores were higher when compared to scores at Pre-Control and Post-Control. Anecdotally, improvements in balance and posture were the most commonly reported changes in participants following the EAA-only intervention and the GaitWay intervention. A secondary purpose was to quantify changes in motor skill proficiency before and after 1 year of EAA and brain-building activities with a subset of youth with ND who participated in the primary analyses (follow-up analyses). With these participants, scores were higher at Post-GaitWay when compared to Post-Washout, and scores were maintained for one additional year with a continuation of the GaitWay program during this period. Anecdotally, caregivers most commonly reported that enhanced coping skills and improvements in balance and posture were observed following the 1-year GaitWay intervention. These results should be helpful to clinicians and healthcare professionals who implement therapeutic strategies to improve motor skill proficiency in youth with ND. As the prevalence and efficacy of these programs increase, there may be a greater demand for the combination of EAT and brain building activities to be recognized by health insurance providers. This may allow for these programs to become more accessible and affordable.

The inherent nature of EAT may allow for changes in motor skill proficiency to be observed. The interaction with a horse appears to physically, socially, and emotionally benefit children with developmental disabilities. Significant improvements in self-confidence, appropriate social behavior, and communication among children with learning and developmental disabilities have been reported with children participating in EAT (39, 40). The bond between the rider and horse may play a critical role with psychosocial health and reducing anxiety and stress. Indeed, behavioral difficulties, activity limitations, environmental obstacles, and personal factors (e.g., attitude, willingness to participate) affect a child's ability to engage in everyday activities (41). With activity that can improve mental health by improving self-concept, social participation, and communication, children will more likely remain, and continue to, engage in that activity (42). The repeated bouts of activity will only further the physical and mental health of those who participate in the intervention (42).

During EAT, the horse's foot fall and muscular movement causes a rhythmic three-dimensional movement of the rider's pelvis and necessitates that the rider engage their musculature to maintain the body's equilibrium (43). Thus, the rider's pelvis motions while horseback riding at a walking pace are similar to those exhibited while walking on a level surface in children (27). As such, these motions serve as atypical activators of skeletal muscle and may, in turn, elicit increases in muscle strength of the rider, particularly at the abdomen, lower back, pelvis, and legs (44). Increasing the strength of skeletal muscles in these areas of the body is crucial to improve abnormal balance and gait dysfunction (45). As balance and gait are needed to properly execute many gross motor skills, the ability for the participant to properly sequence and organize physical tasks, including those for balance, gait and strength, is optimized when EAT is combined with the brain-building activities that are performed consistently and as directed throughout the GaitWay program. Reduced muscle strength is a feature of several forms of ND, including ASD (46), IDD (47), and ADHD (48). An increase in muscle weakness is one of many characteristics associated with obesity (49).

In the current study, the average age and BMI measurements for those participants in the primary analyses place the BMI-for-age at the 75th percentile and therefore at a “healthy weight” (33). Five of the 25 participants in this study were classified as overweight or obese (i.e., greater than the 85th percentile) (33), which aligns with reported prevalence rates of overweight and obesity in youth populations with ADHD (9) and ASD (50). The overweight or obese state may have negatively affected performance in gross motor skills, particularly with strength (as evidenced by 5 or less sit-ups performed in 30 s at any time point), in these youth.

The BOT-2 Short Form was used to quantify motor skill proficiency in this study. This assessment has been used to investigate the effects of EAAT with motor competencies in various populations. Ten weeks of EAT, performed once per week (30 min per session), increased total scores by 20.7% in youth with cerebral palsy (CP) (51). Although CP is a neuromuscular disorder, more than 30% of individuals with CP also have IDD (52) and commonly have other associated conditions, including cognitive and behavioral issues, oral motor dysfunction, and sensory and perception impairments (49). Ten weeks of EAA (performed once per week for 45 min per session) also increased total scores by 7.1–17.4% in youth with ASD, depending on the study (39, 53). In the current study, the EAA and GaitWay 8-week interventions elicited an 8.7 and 8.3% increase in motor skill proficiency, respectively. A 12.4% improvement in motor skills were recorded following the 1-year GaitWay intervention. These results appear to complement the results from other studies that include a similar population, intervention, and assessment. It is interesting that larger increases in motor skill scores are observed with those diagnosed with a non-progressive neuromuscular disease (e.g., CP), so these individuals appear to greatly benefit from EAA and similar interventions. However, if participants exhibit one or more forms of ND, a high variability in total scores are often reported [e.g., (39, 51, 53)]. Large variability in scores was also observed in the present study.

It is well-known that youth with ND inherently experience a delay in motor skills (2). Any improvements gained in motor skills following an intervention could also be delayed. Alternatively, a therapeutic intervention may elicit latent effects with regards to motor skill improvements in youth with ND. For example, Pan et al. found that after a multi-week therapeutic intervention, children with ASD may continue to experience an increasing trend of improvements with select motor skills measured via the BOT-2 (54). In the present study, although not statistically significant, there was a 2.1% improvement in total scores during the washout block (which followed the EAA-only intervention), and a 0.5% increase during the initial control block. The observed improvement following the washout block may be partially due to the carryover benefits in motor skills obtained by the participants during the EAA-only intervention.

Some of the common anecdotal responses from caregivers in this study were similar to those reported in other studies. In youth with ASD, a multi-week EAA intervention can lead to positive changes in mood (55), an improved ability to learn (56), and a more calming behavior, enhanced focus and improved communication (53). In the present study, physiological changes were observed mostly after the EAA-only intervention, with minimal reporting of behavioral changes (see Table 5). However, the GaitWay intervention greatly increased the number of anecdotally-reported physiological and psychosocial improvements, including those observed at home and in a school setting. After 1 year of participating in the GaitWay program, most participants experienced physical, behavioral, and academic improvements (see Table 6). Indeed, long term (i.e., 6 months) of EAA alone can elicit significant improvements in social and communication behaviors, with enhanced language skills (57). There was a noticeably large range of improvements reported after the GaitWay interventions, rather than a very high number reported with respect to one improvement, possibly due to the variability in the participants.

When examining effect sizes, it appears the intervention order explained 1–23% of the observed variability with the BOT-2 Short Form subtest scores. With regard to overall scores, the intervention order explained 25 and 58% of the variability in the primary analyses and follow-up analyses, respectively. The inter-individual differences between participants, including their age, diagnoses, and severity of symptoms, most likely contribute to the majority of the observed variability found in motor skill scores in the study.

A within-group design was employed in the present study, such that participants served as their own control. They may have resulted in learning effects of the BOT-2 Short Form, which was administered at five time points in the primary analyses. Also, due to the length of the study (32 weeks for the primary analyses, with one additional year for the follow-up analyses), the lack of a concurrent control group makes it difficult to discern if improvements in motor skills were due to inherent, age-related changes. The study design was chosen because each participant, regardless of diagnosis, alternated between interventions with periods that did not include horseback riding-related activities. Another limitation of the present study is that multiple statistical tests were performed, which may result in a type I error. Therefore, the results of this study must be approached with caution, as some of the significant results may be false positives. This would be especially true in the significant results with the follow-up analyses (i.e., greater overall motor skill scores following the GaitWay intervention). However, given the small sample size in this analysis, these data may be treated as pilot data for future long-term studies.

The varying diagnoses of participants in this study, including several forms of ND and associated co-diagnoses, was a limitation in this study. As such, some participants were more mildly affected with motor skill proficiency when compared to others. It would be difficult to extrapolate these results to youth with a specific severity of an ND-related diagnosis. Another limitation was that participants were allowed to consume medications and participate in outside therapies throughout the study. An attempt was made to control for other environmental conditions, including testing in the same location, at the same time of day, with the same number of study personnel present when testing and the parent out-of-sight in a nearby room. However, medications and outside therapies—even under controlled, standard conditions—can add to the variability in responses during motor skill testing. Also, while youth were tested in this study, this sample may not be reflective of adults diagnosed with some ND. With the follow-up analyses, these limitations can also be applied. Because of only 7 participants in this analyses, the small sample size limited the statistical power, and prevented the comparison of BOT-2 Short Form subtest scores between time points. Results in general may therefore not be reflective of actual population parameters.

In this study, overall motor skill proficiency, assessed using the BOT-2 Short Form, increased following a washout period from an original baseline 24 weeks prior, and remained increased following a subsequent period of both EAA and brain building activities. The increase observed after the washout period may be related to delayed, or latent, improvements in motor skills following an earlier EAA-only intervention with this population. Overall motor skills remained elevated for one additional year while participating in a combination of EAA and brain building activities. Results should be approached with caution, however, due to the large variability in scores and weak effect sizes with select fine motor and gross motor tasks, and the absence of a concurrent control group for comparison. Future research should include more physiological testing to complement the assessment of motor competencies, and methods to quantitatively measure the anecdotal data, reported in this study, following a multi-week intervention with EAAT and brain building activities.

## Data Availability Statement

The datasets generated for this study are available on request to the corresponding author.

## Ethics Statement

The studies involving human participants were reviewed and approved by Texas Woman's University Institutional Review Board. Written informed consent to participate in this study was provided by the participants' legal guardian/next of kin.

## Author Contributions

BR was the principal investigator for the project and was responsible for project design, data collection, statistical analysis, and article writing. RD was responsible for project design and data collection. MB and GJ were responsible for data collection. RH was responsible for design, implementation, and intervention delivery of the brain-building program. EL was responsible for participant recruitment, study design, and EAA intervention delivery. DN was responsible for statistical analysis and article writing.

### Conflict of Interest

The authors declare that the research was conducted in the absence of any commercial or financial relationships that could be construed as a potential conflict of interest.

## References

[1] Mullin A Gokhale A Moreno-De-Luca A Sanyal S Waddington J Faundez V. Neurodevelopmental disorders: mechanisms and boundary definitions from genomes, interactomes and proteomes. Transl Psychiatry. (2013) 3:e329. 10.1038/tp.2013.108 24301647 PMC4030327

[2] American Psychiatric Association Diagnostic and Statistical Manual of Mental Disorders. 5th ed. Washington, DC: American Psychiatric Publishing (2013).

[3] Boyle C Boulet S Schieve L Cohen R Blumberg S Yeargin-Allsopp M. Trends in the prevalence of developmental disabilities in US children, 1997-2008. Pediatrics. (2011) 127:1034–42. 10.1542/peds.2010-2989 21606152

[4] Hansen B Oerbeck B Skirbekk B Petrovski B Kristensen H. Neurodevelopmental disorders: prevalence and comorbidity in children referred to mental health services. Nord J Psychiatry. (2018) 72:285–91. 10.1080/08039488.2018.1444087 29488416

[5] Bishop J Pangelinan M. Motor skills intervention research of children with disabilities. Res Dev Disabil. (2018) 74:14–30. 10.1016/j.ridd.2017.11.002 29366922

[6] Physical Activity Guidelines Advisory Committee Physical Activity Guidelines Advisory Committee Scientific Report. Washington, DC: U Department of Health and Human Services (2018). Available online at: https://health.gov/paguidelines/second-edition/report/ (accessed October 1, 2019).

[7] Healy S Aigner CJ Haegele JA. Prevalence of overweight and obesity among US youth with autism spectrum disorder. Autism. (2019) 23:1046–50. 10.1177/1362361318791817 30101597

[8] Agranat-Meged A Deitcher C Goldzweig G Leibenson L Stein M Galili-Weisstub E. Childhood obesity and attention deficit/hyperactivity disorder: a newly described comorbidity in obese hospitalized children. Int J Eat Disord. (2005) 37:357–9. 10.1002/eat.20096 15856493

[9] Hölcke M Marcus C Gillberg C Fernell E. Paediatric obesity: a neurodevelopmental perspective. Acta Paediatr. (2008) 97:819–21. 10.1111/j.1651-2227.2008.00816.x 18430075

[10] Mårild S Gronowitz E Forsell C Dahlgren J Friberg P. A controlled study of lifestyle treatment in primary care for children with obesity. Pediatr Obes. (2013) 8:207–17. 10.1111/j.2047-6310.2012.00105.x 23172847

[11] Guo S Wu W Chumlea W Roche A. Predicting overweight and obesity in adulthood from body mass index values in childhood and adolescence. Am J Clin Nutr. (2002) 76:653–8. 10.1093/ajcn/76.3.653 12198014

[12] Whitaker R Wright J Pepe M Seidel K Dietz W. Predicting obesity in young adulthood from childhood and parental obesity. N Engl J Med. (1997) 337:869–73. 10.1056/NEJM199709253371301 9302300

[13] Cortese S Ramos Olazagasti M Klein R Castellanos F Proal E Mannuzza S. Obesity in men with childhood ADHD: a 33-year controlled, prospective, follow-up study. Pediatrics. (2013) 131:e1731–8. 10.1542/peds.2012-0540 23690516 PMC4074659

[14] Matthews CE Chen KY Freedson PS Buchowski MS Beech BM Pate RR. Amount of time spent in sedentary behaviors in the United States, 2003-2004. Am J Epidemiol. (2008) 167:875–81. 10.1093/aje/kwm390 18303006 PMC3527832

[15] Carson V Hunter S Kuzik N Wiebe S Spence J Friedman A. Systematic review of physical activity and cognitive development in early childhood. J Sci Med Sport. (2016) 19:573–8. 10.1016/j.jsams.2015.07.011 26197943

[16] Hinkley T Teychenne M Downing K Ball K Salmon J Hesketh K. Early childhood physical activity, sedentary behaviors and psychosocial well-being: a systematic review. Prev Med. (2014) 62:182–92. 10.1016/j.ypmed.2014.02.007 24534461

[17] Janssen I LeBlanc A. Systematic review of the health benefits of physical activity and fitness in school-aged children and youth. Int J Behav Nutr Phys Act. (2010) 7:40. 10.1186/1479-5868-7-40 20459784 PMC2885312

[18] Mangerud W Bjerkeset O Lydersen S Indredavik M. Physical activity in adolescents with psychiatric disorders and in the general population. Child Adolesc Psychiatry Ment Health. (2014) 8:2. 10.1186/1753-2000-8-2 24450542 PMC3914726

[19] Zeng N Ayyub M Sun H Wen X Xiang P Gao Z. Effects of physical activity on motor skills and cognitive development in early childhood: a systematic review. Biomed Res Int. (2017) 2017:2760716. 10.1155/2017/2760716 29387718 PMC5745693

[20] Stodden D Langendorfer S Roberton M. The association between motor skill competence and physical fitness in young adults. Res Q Exerc Sport. (2009) 80:223–9. 10.1080/02701367.2009.10599556 19650387

[21] Williams H Pfeiffer K O'Neill J Dowda M McIver K Brown W. Motor skill performance and physical activity in preschool children. Obesity. (2008) 16:1421–6. 10.1038/oby.2008.214 18388895

[22] Rinehart N Jeste S Wilson R. Organized physical activity programs: Improving motor and non-motor symptoms in neurodevelopmental disorders. Dev Med Child Neurol. (2018) 60:856–7. 10.1111/dmcn.13962 29963691 PMC7334028

[23] Howells K Sivaratnam C May T Lindor E McGillivray J Rinehart N. Efficacy of group-based organised physical activity participation for social outcomes in children with autism spectrum disorder: a systematic review and meta-analysis. J Autism Dev Disord. (2019) 49:3290–308. 10.1007/s10803-019-04050-9 31102193

[24] Rigby B Grandjean P. The efficacy of equine-assisted activities and therapies on improving physical function. J Altern Complement Med. (2016) 22:9–24. 10.1089/acm.2015.0171 26654868

[25] Quint C Toomey M Powered saddle and pelvic mobility. An investigation into the effects on pelvic mobility of children with cerebral palsy of a powered saddle which imitates the movements of a walking horse. Physiotherapy. (1998) 84:376–84. 10.1016/S0031-9406(05)61458-7

[26] Bertoti DB. Effect of therapeutic horseback riding on posture in children with cerebral palsy. Phys Ther. (1988) 68:1505–12. 10.1093/ptj/68.10.1505 3174832

[27] Garner B Rigby B. Human pelvis motions when walking and when riding a therapeutic horse. Hum Mov Sci. (2015) 39:121–37. 10.1016/j.humov.2014.06.011 25436916

[28] Giagazoglou P Arabatzi F Dipla K Liga M Kellis E. Effect of a hippotherapy intervention program on static balance and strength in adolescents with intellectual disabilities. Res Dev Disabil. (2012) 33:2265–70. 10.1016/j.ridd.2012.07.004 22853887

[29] Jang B Song J Kim J Kim S Lee J Shin HY. Equine-assisted activities and therapy for treating children with attention-deficit/hyperactivity disorder. J Altern Complement Med. (2015) 21:546–53. 10.1089/acm.2015.0067 26167851

[30] Ajzenman H Standeven J Shurtleff T. Effect of hippotherapy on motor control, adaptive behaviors, and participation in children with autism spectrum disorder: a pilot study. Am J Occup Ther. (2013) 67:653–63. 10.5014/ajot.2013.008383 24195899

[31] Del Rosario-Montejo O Molina-Rueda F Muñoz-Lasa S Alguacil-Diego I Effectiveness of equine therapy in children with psychomotor impairment. Neurologia. (2015) 30:425–32. 10.1016/j.nrl.2013.12.023 24656851

[32] Miller LJ Nielsen DM Schoen SA Brett-Green BA. Perspectives on sensory processing disorder: a call for translational research. Front Integr Neurosci. (2009) 3:22. 10.3389/neuro.07.022.2009 19826493 PMC2759332

[33] Centers for Disease Control and Prevention. Defining Childhood Obesity. US Department of Health and Human Services (2018). Available online at: https://www.cdc.gov/obesity/childhood/defining.html (accessed October 1, 2019).

[34] Bruininks R Bruininks B. Bruininks-Oseretsky Test of Motor Proficiency. 2nd ed. Minneapolis, MN: NCS Pearson (2005).

[35] Deitz J Kartin D Kopp K. Review of the Bruininks-Oseretsky test of motor proficiency, second edition (BOT-2). Phys Occup Ther Pediatr. (2007) 27:87–102. 10.1080/J006v27n04_06 18032151

[36] Jírovec J Musálek M Mess F. Test of motor proficiency second edition (BOT-2): compatibility of the complete and Short Form and its usefulness for middle-age school children. Front Pediatr. (2019) 7:153. 10.3389/fped.2019.00153 31065548 PMC6489893

[37] Fransen J D'Hondt E Bourgois J Vaeyens R Philippaerts R Lenoir M. Motor competence assessment in children: convergent and discriminant validity between the BOT-2 Short Form and KTK testing batteries. Res Dev Disabil. (2014) 35:1375–83. 10.1016/j.ridd.2014.03.011 24713517

[38] Dennison G Dennison P Menu for the Brain Gym^®^ Course. Ventura, CA: Edu-Kinesthetics Inc (2017).

[39] Gabriels R Pan Z Dechant B Agnew J Brim N Mesibov G. Randomized controlled trial of therapeutic horseback riding in children and adolescents with autism spectrum disorder. J Am Acad Child Adolesc Psychiatry. (2015) 54:541–9. 10.1016/j.jaac.2015.04.007 26088658 PMC4475278

[40] Macauley B Gutierrez K The effectiveness of hippotherapy for children with language-learning disabilities. Commun Disord Q. (2004) 25:205–17. 10.1177/15257401040250040501

[41] Majnemer A Shevell M Law M Poulin C Rosenbaum P. Level of motivation in mastering challenging tasks in children with cerebral palsy. Dev Med Child Neurol. (2010) 52:1120–6. 10.1111/j.1469-8749.2010.03732.x 20646031

[42] Kipp LE. Psychosocial aspects of youth physical activity. Pediatr Exerc Sci. (2017) 29:35–8. 10.1123/pes.2017-0005 28271811

[43] MacPhail HEA Edwards J Golding J Miller K Mosier C Zwiers T Trunk postural reactions in children with and without cerebral palsy during therapeutic horseback riding. Pediatr Phys Ther. (1998) 10:143–7.

[44] Benda W McGibbon N Grant K. Improvements in muscle symmetry in children with cerebral palsy after equine-assisted therapy (hippotherapy). J Altern Complement Med. (2003) 9:817–25. 10.1089/107555303771952163 14736353

[45] Chvatal S Ting L. Common muscle synergies for balance and walking. Front Comput Neurosci. (2013) 7:48. 10.3389/fncom.2013.00048 23653605 PMC3641709

[46] Kern J Geier D Adams J Troutman M Davis G King P. Handgrip strength in autism spectrum disorder compared with controls. J Strength Cond Res. (2013) 27:2277–81. 10.1519/JSC.0b013e31827de068 23880656

[47] Dey A Bhowmik K Chatterjee A Chakrabarty P Sinha S Mukhopadhyay K. Down syndrome related muscle hypotonia: association with COL6A3 functional SNP rs2270669. Front Genet. (2013) 4:57. 10.3389/fgene.2013.00057 23626599 PMC3631610

[48] Jeoung B. The relationship between attention deficit hyperactivity disorder and health-related physical fitness in university students. J Exerc Rehabil. (2014) 10:367–71. 10.12965/jer.140175 25610821 PMC4294439

[49] Ehrman JK Gordon PM Visich PS Keteyian SJ Clinical Exercise Physiology. Champaign, IL: Human Kinetics (2019).

[50] Kamal Nor N Ghozali A Ismail J. Prevalence of overweight and obesity among children and adolescents with autism spectrum disorder and associated risk factors. Front Pediatr. (2019) 7:38. 10.3389/fped.2019.00038 30842939 PMC6391908

[51] Champagne D Corriveau H Dugas C. Effect of hippotherapy on motor proficiency and function in children with cerebral palsy who walk. Phys Occup Ther Pediatr. (2017) 37:51–63. 10.3109/01942638.2015.1129386 26930110

[52] Cans C Guillem P Arnaud C Baille F Chalmers J McManus V Prevalence and characteristics of children with cerebral palsy in Europe. Dev Med Child Neurol. (2002) 44:633–40. 10.1017/S0012162201002675 12227618

[53] Gabriels R Agnew J Holt K Shoffner A Zhaoxing P Ruzzano S Pilot study measuring the effects of therapeutic horseback riding on school-age children and adolescents with autism spectrum disorders. Res Autism Spectr Disord. (2012) 6:578–88. 10.1016/j.rasd.2011.09.007

[54] Pan CY Chu CH Tsai CL Sung MC Huang CY Ma WY. The impacts of physical activity intervention on physical and cognitive outcomes in children with autism spectrum disorder. Autism. (2017) 21:190–202. 10.1177/1362361316633562 27056845

[55] Kern J Fletcher C Garver C Mehta J Grannemann B Knox K. Prospective trial of equine-assisted activities in autism spectrum disorder. Altern Ther Health Med. (2011) 17:14–20. 22164808

[56] Ward S Whalon K Rusnak K Wendell Paschall N. The association between therapeutic horseback riding and the social communication and sensory reactions of children with autism. J Autism Dev Disord. (2013) 43:2190–8. 10.1007/s10803-013-1773-3 23371511

[57] Gabriels R Pan Z Guérin N Dechant B Mesibov G. Long-term effect of therapeutic horseback riding in youth with autism spectrum disorder: a randomized trial. Front Vet Sci. (2018) 5:156. 10.3389/fvets.2018.00156 30062099 PMC6054954

